# Microstructure, Mechanical and Electrical Properties of Hybrid Copper Matrix Composites with Fe Microspheres and rGO Nanosheets

**DOI:** 10.3390/molecules27196518

**Published:** 2022-10-02

**Authors:** Xinjiang Zhang, Meng He, Yongzhong Zhan, Wenchao Yang, Kaifeng Wu

**Affiliations:** 1 School of Materials Science and Engineering, Yancheng Institute of Technology, Yancheng 224002, China; 2 Guangxi Key Laboratory of Processing for Non-Ferrous Metals and Featured Materials, Guangxi University, Nanning 530004, China

**Keywords:** hybrid composites, powder metallurgy, secondary phase, mechanical properties

## Abstract

Copper matrix composites have a wide application as magnetic conductive materials, electromagnetic materials, electrical discharge machining materials, etc. Such materials are expected to have a good combination of excellent electrical conductivity and good mechanical strength. In this work, micro/nano hybrid reinforcements with Fe microspheres and reduced graphene oxide (rGO) nanosheets were developed for copper matrix composites. The rGO/Fe/Cu powders were firstly wet-mixed and then densified by the vacuum hot-pressing sintering to obtain the bulk compacts. Microstructure, electrical conductivity and mechanical properties of such compacts were investigated. Microstructural result of as-sintered compacts shows that the Fe microspheres could distribute in the matrix uniformly, and rGO nanosheets exhibit both agglomerated and dispersed states. The grain size of Cu matrix decreased with the increase of the rGO content. Hardness, compression and tensile 0.2% yield strength of the as-sintered compacts were improved evidently by the addition of the hybrid Fe/rGO, comparing with pure Cu and single Fe-added composites. However, a lower electrical conductivity appeared in the more rGO-added composites, but still reached more than 33.0% international annealing copper standard (IACS). These performance change could be sought in the spatially geometrical distribution and characteristics of such micro/nano Fe/rGO hybrid addition, and the relevant mechanisms were discussed.

## 1. Introduction

Copper (Cu) is quite popular as a structural and functional engineering metal due to its high electrical and thermal conductivity, good ductility and high corrosion resistance, etc. [1]. However, its low mechanical strength has limited its range of application [2,3]. Many efforts have been made by introducing a secondary phase into Cu matrix for achieving the excellent electrical and thermal conductivity as well as high strength and plasticity. Thereinto, some transitional metal micro-particles (Nb [4,5], Ag [6,7], Fe [8,9,10] and Cr [11]) are attempted and added into Cu to adjust the microcomposites, and further adjust the structural characteristic of materials by means of various alloying and preparation methods. In these Cu-based microcomposites, the specific advantage of Cu–Fe system is the relatively low cost of Fe compared with both of Cu–Nb and Cu–Ag systems [9,10]. However, high Fe contents significantly reduce the conductivity of copper matrix. Apart from the microcomposites with the single Fe micro-particles, the current design practice for optimizing performance has been typically realized by introducing the alloying elements and deformation [12,13,14,15,16]. Song et al. [17] processed Cu–Fe–Ag and Cu–Fe–Cr microcomposites and found that Cu–Fe–Ag microcomposites exhibited finer filament and higher strength. The addition of rare earth not only considerably refines the microstructure but also improves the conductivity of Cu–6 wt.% Fe microcomposite [16]. Still, the new reinforcing mode for copper matrix composites (CMCs) are continuously explored for the requirement of high-performance in order to extend their application.

Besides the single metal micro-particles, the hybrid addition of micro-sized reinforcements (particles and/or whiskers) is another reinforcing method in CMCs to achieve more excellent properties (hardness, strength, electrical conductivity, friction coefficient) by a cooperative strengthening effect of different reinforcements rather than by increasing the additive content [18,19,20,21,22]. Typically, the advantages of hybrid reinforcements composed of particles and/or whiskers have been clearly confirmed in CMCs. Further, the micro/nano hybrid metal matrix composites simultaneously incorporating nano-dimensional fillers and conventional micro-scaled fillers have been recently developed as a novel reinforcing mode [2,23]. The mixing of micro/nano constituents (ceramics particles, carbon fiber (Cf), carbon nanotubes (CNTs), etc.) enables the preparation of composites with new or improved properties due to the synergistic effects. For such reinforcing mode, various hybrid metal matrix composites have been developed for such applications including SiC/Cu [2], TiB_2_/CNTs/Cu [24], SiC/Fe [25], SiC/TiB_2_/Al [26], and so on. Using micro/nano reinforcements in Cu matrix could improve elastic modulus, strength, hardness and tribological properties better than that by using single-scale reinforcement.

Due to superior properties such as super electron mobility (200,000 cm^2^V^−1^s^−1^ [27]), large surface area (2600 m^2^g^−1^ [28]) and extraordinary fracture strength (125 GPa [29]), a two-dimensional layered graphene has attracted tremendous attention as a novel nanoscale reinforcing filler for CMCs. The single graphene was recently introduced into Cu matrix to improve the mechanical performance [30,31,32]. Qiao et al. [30] successfully fabricated three-dimensional interpenetrating network graphene/Cu composites by rolling and sintering. Benefitting from such structure, the composites exhibited an enhanced tensile strength (354 MPa), while maintaining good ductility (16.5% elongation) and robust conductivity (98% IACS). The tensile strength of hierarchical layered reduced graphene oxide (rGO)/Cu composites was much higher than that of the pure Cu [31]. Similar to carbon nanotubes (CNTs), the nano-sized graphene is also easy to agglomerate and shows poor wettability and weak graphene/Cu interface bonding, which hinders the further development to increase its strengthening efficiency. But above all, it is a challenge to obtain uniformly dispersed graphene with high contents in CMCs, and the nano-scale graphene with limited volume fractions always lead to limited increase in mechanical properties [33].

Considering the micro/nano hybrid structure, the conventional Fe microspheres and novel lamellar rGO nanosheets as a micro/nano hybrid reinforcing methodology was applied to fabricate novel CMCs in this study. Such hybrid CMCs are expected as the promising conductor materials. Such micro/nano hybrid CMCs were fabricated by vacuum hot-pressing sintering (HPS) process. In addition, the phase structure, microstructure, electrical conductivity and mechanical properties were studied in detail and compared with those of a series of HPS-fabricated pure copper and single Fe microspheres added composites.

## 2. Results and Discussion

### 2.1. Structure of rGO/Fe/Cu Mixture Powders

**Figure 1:**
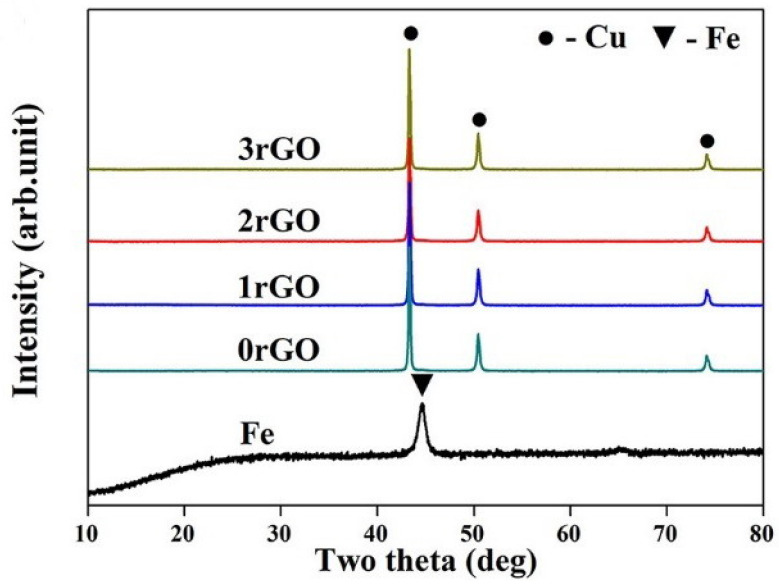
XRD patterns of the mixture powders and original Fe.

**Figure 2:**
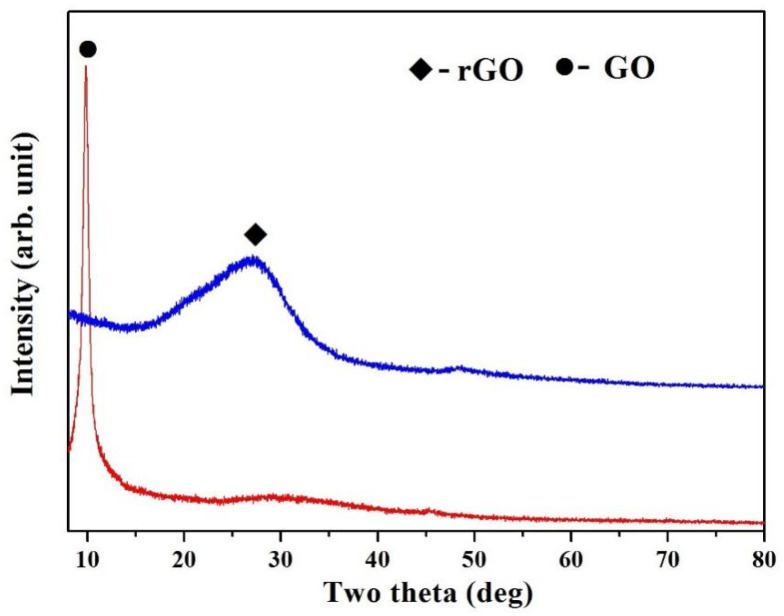
XRD patterns of rGO and GO.

XRD patterns of the mixture powders and original Fe are shown in Figure 1. For the mixture powders (Figure 1), three characteristic peaks at 2θ = 43.3°, 50.4°, 74.2° were corresponded to (111), (200) and (220) crystallographic planes of face-centered cubic (fcc) structured Cu phase, respectively. Compared with the characteristic peaks of original Fe, Fe phase were not detected in the mixture powders, possibly due to the low content of Fe and the strong peaks of Cu. The rGO was investigated by XRD together with starting GO, as shown in Figure 2. A strong peak of GO was found at 2θ = 9.8°, indicating the formation of intercalated water moieties and oxygen functionalities groups of GO [34]. For rGO in the mixture powders, the peak at 2θ = 9.8° disappeared completely, and a broad (002) characteristic peak appeared at 2θ = 27.1°. It indicated the removal of some oxygen-containing functional groups and the formation of graphite-like structure in rGO.

**Figure 3:**
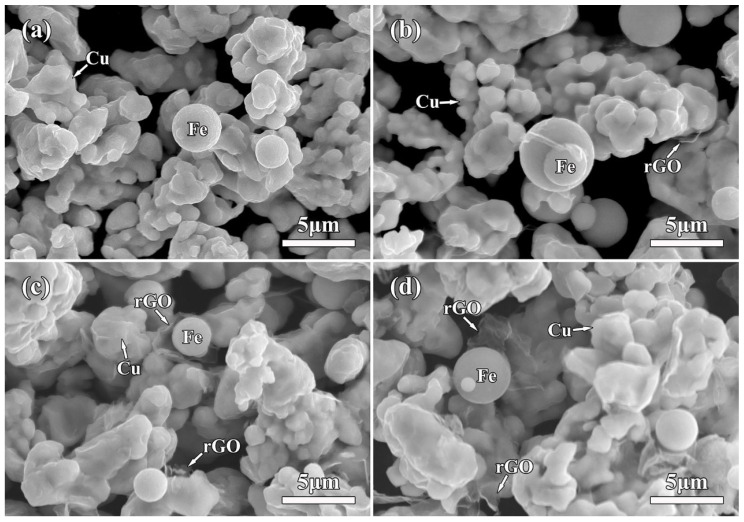
Low-magnification SEM micrographs of the as-prepared mixture powders: (**a**) 0rGO, (**b**) 1rGO, (**c**) 2rGO, (**d**) 3rGO.

**Figure 4:**
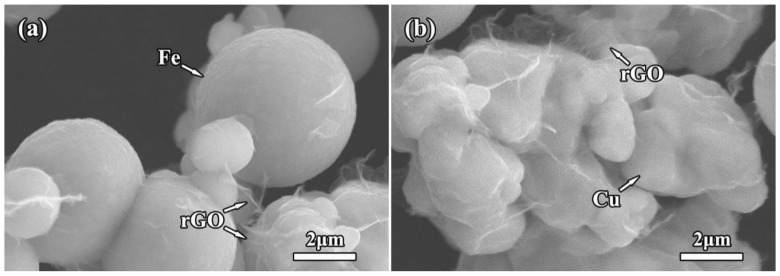
High-magnification SEM micrographs showing the rGO nanosheets coated on the surface of Fe microspheres (**a**) and Cu particles (**b**) in the mixture powders.

**Figure 5:**
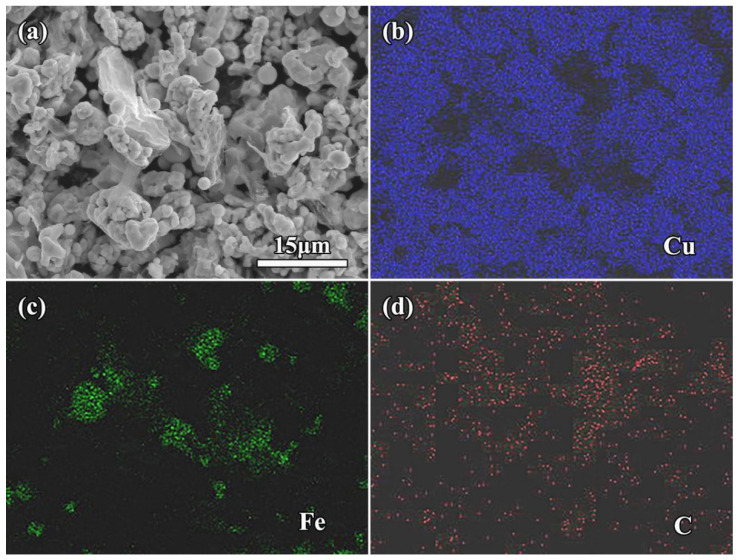
(**a**) SEM-BSE micrograph of the as-prepared 3rGO mixture powders; (**b**,**c**) X-ray dot maps of Cu (**b**), Fe (**c**) and C (**d**) distribution.

Microscopic observation with low magnifications shows clearly the abundant Fe microspheres and Cu particles in the mixture powder. Figure 3 shows the SEM micrographs of rGO/Fe/Cu mixture powders. Obviously, most of Fe microspheres were independent from each other, indicating the uniform mixture of Fe/Cu powders using the wet-mixing process. Interestingly, layered rGO nanosheets also appeared in the matrix. Moreover, 3rGO mixture powders had more rGO nanosheets. The rGO nanosheets without obvious agglomeration were homogeneously distributed into Fe/Cu powders, indicating that the layered rGO nanosheets had been randomly dispersed into Fe/Cu powders. Higher-magnification SEM observation clearly showed the rGO nanosheets on the surface of Fe microspheres (Figure 4a), being related to the good bonding between GO and Fe microspheres before reduction. Moreover, it can be found that the rGO nanosheets coated tightly on the Cu surface (Figure 4b). The two-dimensional nano-layer rGO had the high specific surface energy and adsorbed easily on other particles. Meyer et al. [35] confirmed that the corrugation was the unique feature of graphene nanosheets and decreased or even disappeared with the increasing of number of graphene layers. The rGO nanosheets with few layers had good transparency and obvious wrinkle. In order to further confirm the existence of Fe microspheres and rGO nanosheets, EDS results of 3rGO mixture powders are presented in Figure 5. Obviously, the element Fe mainly concentrated in the spherical particles, and the element C mainly concentrated in the layered rGO structure. It further indicated that the rGO nanosheets and Fe microspheres were successfully mixed with Cu powders.

### 2.2. Structure of the Sintered Bulk Compacts

**Figure 6:**
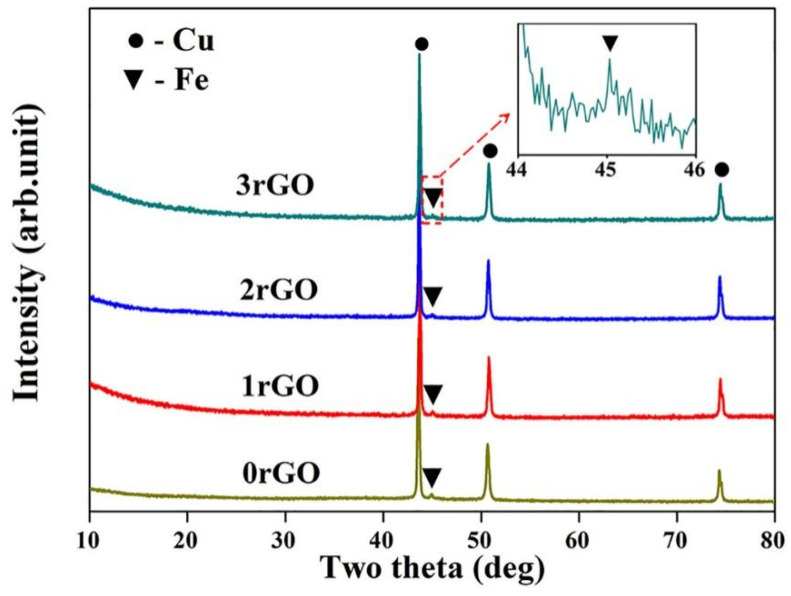
XRD patterns of the sintered bulk compacts. The inset shows the diffraction peak of Fe phase in the bulk compacts.

After sintering of rGO/Fe/Cu mixture powders, XRD patterns of the sintered bulk compacts are shown in Figure 6. The presence of XRD peaks at 2θ of 43.3°, 50.4° and 74.2° revealed the existence of FCC structured Cu phase in the sintered bulk compacts, which were in agreement with XRD results of the mixture powders (Figure 1). Meanwhile, a tiny peak was recognizable beside Cu peak at 2θ = 43.3° in XRD patterns of all samples, which was corresponded to the diffraction peak of αFe, as seen from the inset in Figure 6. Those above results indicated that Cu and Fe phases existed in the sintered bulk compacts. Moreover, it should be noted that no characteristic peaks of rGO were detected, possibly due to the strong peaks of Cu. During the sintering of rGO/Fe/Cu mixture powders, the other new crystalline products were not found and formed. Based on binary phase diagrams from Cu–Fe and Cu–C systems [36], no binary compounds could be formed between them. In the Cu–Fe alloys prepared by the sintering and casting methods, both Cu and Fe phases were found and further confirmed no formation of Cu–Fe compound [8,10]. The sintering of Cu/rGO mixture powders showed that both Cu and graphite-like C phases existed in their bulk compacts [20]. The structures of Fe/Cu/FeC_0.05_ (wt.%) diffusion couple annealed at 1000 °C for 240 h indicated the existence of Fe, Cu and graphite phases [36]. The ternary phases (Fe, Cu and carbon) could coexist in Cu–Fe–C system at different temperatures (850 °C, 925 °C and 1050 °C) based on the experimental and calculated data [36,37]. No obvious compound could be generated during the sintering process of rGO/Fe/Cu mixture powders at 900 °C for 2 h.

**Figure 7:**
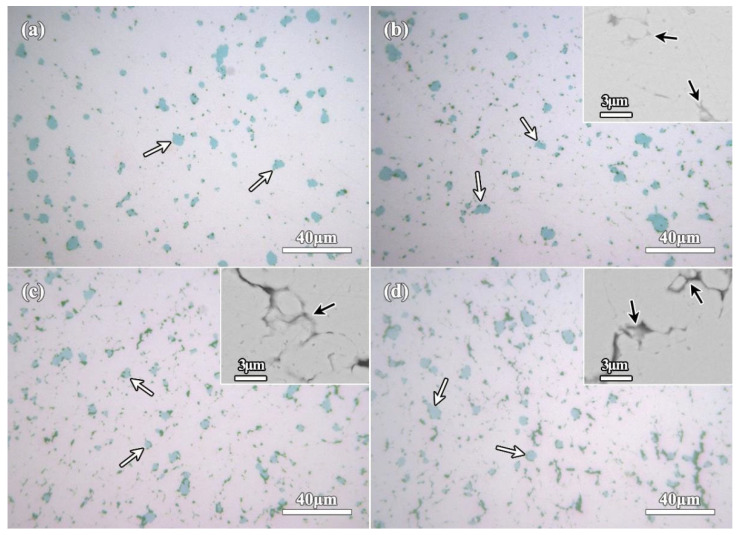
OM micrographs of the sintered compacts showing the dispersed Fe microspheres (marked by white arrows): (**a**) 0rGO, (**b**) 1rGO, (**c**) 2rGO, (**d**) 3rGO. The insets show BSE-SEM micrographs of rGO nanosheets agglomeration (marked by black arrows) in the sintered compacts.

**Figure 8:**
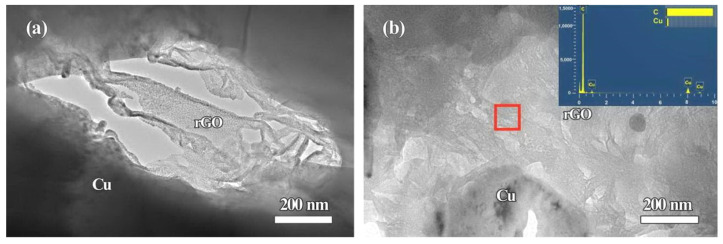
TEM images of rGO nanosheets in the sintered compacts 1rGO (**a**) and 3rGO (**b**) samples. The inset shows EDS result of rGO nanosheets.

Figure 7 shows OM micrographs of the sintered compacts of rGO/Fe/Cu mixture powders. As seen from the microscopic observation in Figure 7, abundant Fe microspheres (marked by white arrows) could be found in each sample. Especially, its distribution was not affected by the different rGO contents and exhibited the uniformity in Cu matrix. The further BSE-SEM observations show the dispersed rGO nanosheets (marked by black arrows) in Cu matrix, as seen the insets in Figure 7. With the increase of rGO content, more irregular rGO were observed. For further characterizing rGO nanosheets, the sintered compacts (1rGO and 3rGO samples) were observed by TEM, and the corresponding results are shown in Figure 8. Interestingly, the layered rGO nanosheets are further confirmed, and mainly exhibit the irregular lamellar shape in the microstructure. Comparing with 1rGO sample, the low G aggregation distributed in more rGO nanosheets added 3rGO sample, indicating the easier adsorption of rGO nanosheets in higher content. Further examination revealed that there is no interfacial reaction at the rGO/Cu interfaces. Thus, it can be deduced that their interfaces are stable between rGO nanosheets and Cu matrix. The microstructural formation process of such bulk compacts involves the wet-mixing and sintering of the rGO/Fe/Cu mixture powders. As starting materials, Cu/Fe powders were added into GO dispersed solution. As such wet dispersing and mixing liquid-solid material, the mechanical stirring realized effectively Cu/Fe/GO mixing in the slurry. The final process of air-drying and N_2_H_4_·H_2_O reduction, the rGO nanosheets were stored in the mixture powders. From these processes, the rGO nanosheets and Fe microspheres were well-distributed with Cu powders (Figure 3). During the high-temperature sintering at 900 °C, the solid-state nucleation and growth of Cu matrix based on Cu atoms diffusion. During the sintering process, the pressure further promoted the densification of the mixture powders. The well-distributed rGO/Fe in the mixture powders were fixed in the Cu matrix, which distributed uniformly in the compacts.

**Figure 9:**
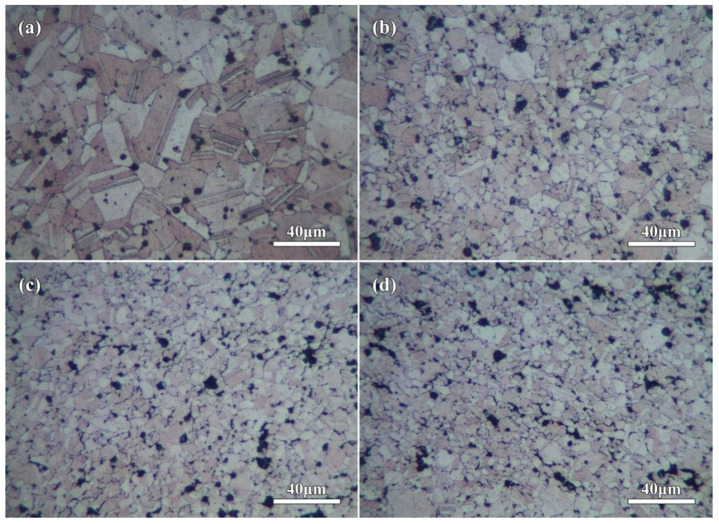
OM micrographs of the corroded compacts showing the refined Cu grains in rGO nanosheets added samples: (**a**) 0rGO, (**b**) 1rGO, (**c**) 2rGO, (**d**) 3rGO.

For further observing the grain characteristic of Cu matrix, OM micrographs of the corroded sintered compacts are shown in Figure 9. Obviously, the grain structure of Cu matrix in Cu–4 wt.%Fe compact (0rGO sample, Figure 9a) exhibited the form of twinning and polygonal grains with visible grain boundary. The uniform circular or quasi-circular black Fe microspheres could be observed in the Cu grains and grain boundaries. Comparing with the micrographs of original dendritic Cu and spherical Fe powders, the morphology of Cu powders was changed obviously before and after sintering, and the growth of Cu grains occurred during the sintering. For the rGO-added compacts (Figure 9b–d), the Cu grains become finer, and the increased rGO addition resulted into smaller Cu grains in the compacts. The grain size of Cu matrix was ~30 μm (0rGO sample), which was refined to ~8 μm (3rGO sample). It indicated that the introduction of rGO nanosheets could significantly refine Cu matrix and prevent the abnormal growth of Cu grains. However, with the increasing of rGO nanosheets, the rGO nanosheets at grain boundaries increased significantly due to the nanosheets agglomeration. The reaction and interdiffusion between Cu/C atoms were not found from the reports about sintered Cu–rGO and Cu–graphene compacts [20,32]. According to microscopic observation of rGO/Fe/Cu mixture powders (Figure 3 and Figure 4), the layered rGO nanosheets coated and wrapped up Cu particles. They could act as a physical barrier to reduce the chance of touching each other between Cu particles. During the sintering process of rGO-added mixture powders, the heat-activated Cu atoms could not get past these layered rGO nanosheets to restrain the rapid diffusion, and then inhibit the grain growth of Cu matrix. Thus, the rGO nanosheets added compacts formed the refined Cu grains, and more rGO addition had a better refinement effect.

### 2.3. Electrical Conductivity of the Sintered Bulk Compacts

**Figure 10:**
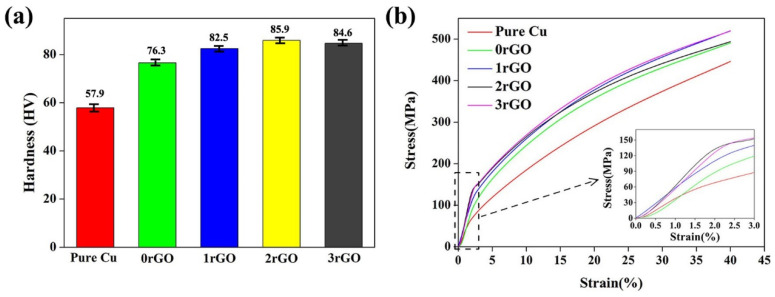
Hardness (**a**) and room-temperature compressive stress-strain curves (**b**) of the sintered pure copper and bulk compacts.

Table 1 lists the electrical conductivity of the sintered bulk compacts. The addition of Fe particles greatly reduced the conductivity of Cu matrix, which was confirmed by the other reports about Cu-Fe alloys [10]. Meanwhile, the conductivity of the compacts decreased further after the introduction of rGO, and which reduced with the accumulation of rGO contents. This result was consistent with the similar observations made on the sintered Cu-graphene systems [38]. In addition, it has been reported that the carbon nanotubes can lead to decreased conductivity of Cu matrix [39]. In this work, the electrical conductivity decreased from 36.0% IACS for 0rGO (single Fe microspheres reinforcements) to 33.8% IACS for 3rGO (hybrid Fe microspheres and rGO nanosheets). This level of electrical conductivity makes such compacts competitive with the previously reported CMCs reinforced by single Fe and Nb metal particles, as shown in Table 1. Electrical conductivity of such compacts depends on not only the electrical properties of constituent phases, but also the reinforcements’ content and defects density. The conductivity of Fe was not as good as that of Cu, and the scattering of electron waves is intensified at the Cu/Fe interface to decrease the conductivity. Solid phase sintering can’t guarantee completely the compactness of the composites, broking the continuity in the conductivity path. The rGO addition caused the refined Cu grains (Figure 10). Generally, the grain refinement would cause the increase of interface density in the compacts, which increased the interface scattering resistance [9]. As a result, the conductivity of compacts was reduced with the improvement of the general resistance.

### 2.4. Mechanical Properties of the Bulk Compacts

The hardness comparison of the bulk compacts and sintered pure copper samples are shown in Figure 10a. Under the same sintering process, the hardness of 0rGO compact (4.0 wt.% Fe microspheres addition) increased to 76.3 HV, which was 31.8% higher than the pure copper (57.9 HV), indicating the remarkable hardening of Fe microspheres. After adding hybrid Fe microspheres and rGO nanosheets, the hardness of the bulk compacts was further increased, which reached a maximum (85.9 HV, 2rGO sample) and then drops down (84.6 HV, 3rGO sample). It illustrated the marked hardening of Fe/rGO hybrids to Cu matrix, and the rGO content played an important role in hardening effect of the bulk compacts.

**Figure 11:**
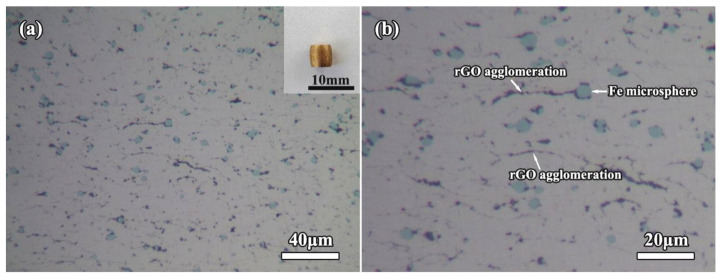
Low- (**a**) and high-magnification (**b**) OM images of the room-temperature deformed 3rGO compact after the compression. The insets show the macrograph of 3rGO compact after compression.

Room-temperature compressive stress-strain curves of the bulk compacts are shown in Figure 10b. From the stress-strain curves, the compressive 0.2% yield strength (σ_0.2_) was calculated to be 88.9 MPa for 0rGO compact and 48.1 MPa for pure copper. Surprisingly, the compressive σ_0.2_ values of compacts further increased from 1rGO sample to 3rGO sample, which were 121.0 MPa (1rGO sample), 125.0 MPa (2rGO sample) and 132.8 MPa (3rGO sample), respectively. The compression test results of the bulk compacts revealed that the hybrid Fe microspheres and rGO nanosheets could act as the efficient strength enhancer. Tan et al. [41] reported the ultimate compressive strength for 186.45 MPa and compressive strain for 39.38% in the 1.0 wt.% graphene added CMCs. All hybrid Fe/rGO added samples did not ruptured under 40% compressive strain, indicating their good compressive deformation capability. Meanwhile, their compressive strength was more than 490 MPa under 39.38% compressive strain, which fully reflected the advantages of hybrid strengthening. To further investigate the deformation evolution of the as-sintered bulk compacts, the deformed microstructure of 3rGO compact was examined, as shown in Figure 11. After the compression, the 3rGO compact was larger in diameter and smaller in height, as shown in the upper right corner of Figure 11a. In its deformed microstructure (Figure 11a), comparing with the no-deformed compact (Figure 7d), the morphology and dispersion of Fe microspheres do not change significantly, but the rGO agglomeration tended to be in the same direction in Cu matrix. The compressed metallographic diagram with a higher magnification is shown in Figure 11b. Some striped rGO agglomeration was almost perpendicular to the direction of force.

**Figure 12:**
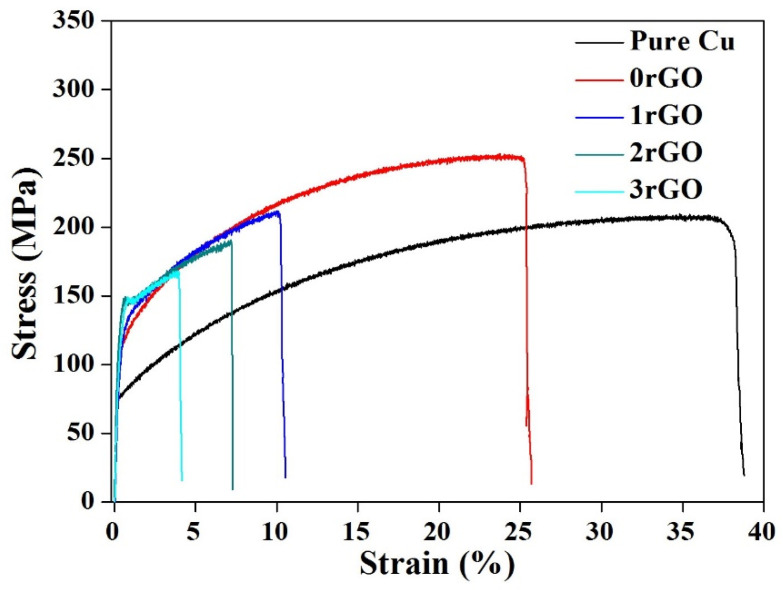
Room-temperature tensile stress-strain curves of the sintered pure copper and bulk compacts.

The representative tensile stress-strain curves of the as-prepared bulk compacts at room temperature are shown in Figure 12. The comparison of obtained tensile 0.2% yield strength (σ_0.2_), ultimate tensile strength (UTS) and elongation values are summarized in Table 2. Under the same sintering process, the tensile σ_0.2_ and UTS of 0rGO compact (single Fe microspheres addition) were 109.2 MPa and 251.3 MPa, respectively, which are higher than that of the pure copper. It indicated the remarkable strengthening of Fe microspheres. This result about Fe strengthening effect in Cu matrix was consistent with the reports on the casted and sintered Cu–Fe alloys [10,40]. After adding hybrid Fe microspheres and rGO nanosheets, the tensile σ_0.2_ value of the bulk compacts is further increased, which reaches a maximum (134.8 MPa, 2rGO sample) and then drops down (129.4 MPa, 3rGO sample), beyond tensile σ_0.2_ values of the unreinforced pure Cu (76.3 MPa) and 0rGO compact (109.2 MPa). However, comparing with 0rGO compact, the tensile UTS values decreased by adding the rGO in the compacts, which were lower values with more rGO addition, as shown in Table 2 and Figure 12. Significantly, this UTS level (210.8 MPa (1rGO sample), 189.8 MPa (2rGO sample)) obtained such novel hybrid Fe/rGO reinforced CMCs competitive with the previously reported CMCs reinforced by single carbon fibers [42], graphene nanoplates [32] and graphene nanoribbons [43]. Moreover, the more rGO addition results in a lower elongation, and the elongation dropped from 25.2% to 3.9% with the content of rGO increased, as shown in Table 2. Many other reports about graphene/Cu composites have produced results essentially in agreement with this phenomenon [32,38].

**Figure 13:**
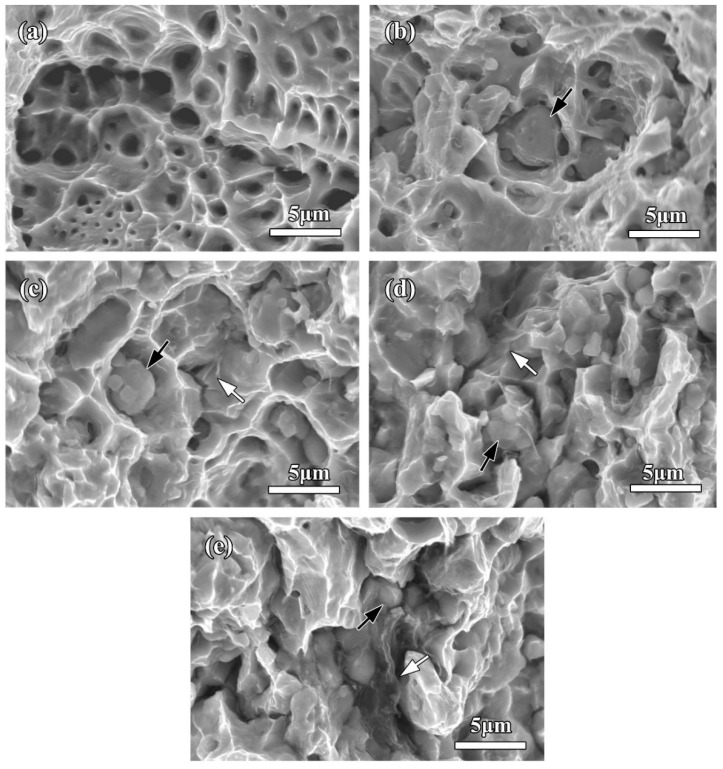
SEM micrographs of tensile fractography of the sintered pure copper and bulk compacts: (**a**) pure copper, (**b**) 0rGO, (**c**) 1rGO, (**d**) 2rGO, (**e**) 3rGO; where the white and black arrows mark the rGO nanosheets and Fe microspheres on the tensile fractography, respectively.

Figure 13a shows SEM image of tensile fractographs of the sintered pure copper. Numerous dimples were observed on the fracture morphology of pure copper and elongated in the loading direction of tensile stress. Such coarse fracture surface gave a clear demonstration of ductile fracture of pure copper under the tensile loading. The tensile fractograph of 0rGO compact (single Fe microspheres addition) is presented in Figure 13b. With the numerous dimples, the broken Fe microspheres could be found on the fracture surfaces. This fractograph indicated the strengthening effect of Fe microspheres. Since the breaking strength of Fe was higher than that of the copper. The Fe microspheres could withstand the load until being broken during the ongoing process of adding tensile force when the compact suffered the tensile stress. In contrast, the dimples obviously decreased for fractured compacts added by hybrid Fe microspheres and rGO nanosheets (Figure 13c–e), resulting in their lower plasticity. In addition, some Fe microspheres had been pulled out from the fracture surfaces. It was especially noteworthy that the rGO nanosheets were observed to be pulled-out or embedded in Cu matrix (marked by white arrows in Figure 13c), suggesting that the stronger rGO/Cu interfacial interaction. When the rGO addition increased to 0.3 wt.%, the rGO/Cu bonding properties became weak due to their weak wettability, thus obvious lamellar structure could be observed in the fracture morphology (Figure 13d,e). Moreover, the fracture surface with a few dimples indicated a changing from ductile fracture to brittle fracture for the fracture mode of compacts.

Based on the above results, the introduction of micro/nano hybrid reinforcements with Fe microspheres and rGO nanosheets improved the compression and tensile σ_0.2_ of Cu matrix, and the strengthening mechanism could be explained from two aspects: spatially geometrical distribution and characteristic of Fe/rGO reinforcements. The hard particle-reinforcements can transfer the load from Cu matrix when CMCs bore the stress [19]. Under the action of external force, Cu matrix with good plasticity was first deformed. Fe microspheres distributed uniformly in Cu matrix (Figure 7). The load transfer could occur from plastic Cu matrix to Fe microspheres (Figure 11 and Figure 13), improving the strength. Further, the Fe microspheres are always pinned to the grain boundaries, which impedes the movement of grain boundaries under stress and then improve the deformation resistance of matrix [9]. From the observation of bulk compacts (Figure 7 and Figure 8), a part of rGO nanosheets displayed the random distribution and different orientation and size, while another part of rGO nanosheets agglomerated in some areas of the matrix, resulting in the formation of laminar structure in the whole microstructure. Under the action of compressive stress, different sized rGO nanosheets could be crimped and folded due to their different orientation, improving the load transfer. Meanwhile, the fragmented and agglomerated rGO were forced by shear flow to form a preferential banded rGO structure along the elongated Cu grains. The gradually straightened rGO could also play a role in penning the dislocation motion. The dislocation pile-up formed near the grain boundaries [44]. The obstacle of dislocation motion increased the critical stress for dislocation glide, leading to an increase of yield strength of the composites. The critical mismatch of coefficient of thermal expansions (CTEs) and elastic moduli among constituent phases (Fe, rGO and Cu matrix) increased the dislocation density in a plastic zone during the large plastic deformation, effectively increase the flow stress upon an applied strain. Cracks occur when the force exceeds the maximum strength of Cu matrix. The load can be transferred to the two-dimensional nanocarbon reinforcements through the action of shear stress at the nanocarbon/Cu interface. The rGO nanosheets have the different spatial distribution in the matrix. Through the load transfer effect of the interface, rGO nanosheets can bear the stress and hinder the cracks propagation. As the loading proceeded, a part of rGO nanosheets was pulled out. The nanoscale rGO existed at the grain boundaries and refined effectively Cu grains (Figure 9). The grain size of Cu matrix decreased with the increase of rGO content by means of hindering the contact between metal particles to prevent abnormal grain growth. It is reasonable to conclude that the strengthening effect is caused by Cu refinement according to Hall–Petch relationship [38]. Although the nanolayer of rGO has the ultra-high strength and elastic modulus, the wettability between copper and rGO is poor. Moreover, the nanocarbon in the matrix separates the interaction between the particles. The bonding between metals is mainly maintained by metal bonds with high bond strength. In spite of rGO contributing to load transfer, the increasing of tensile strength (UTS) is offset by the strength decrease caused by the decreasing of interfacial bonding strength between metal particles. Hence, the UTS increasing is restricted with the increased rGO content. In addition, the agglomeration tendency of rGO nanosheets increased with the increased content, which could not fully exert their performance advantages. The agglomeration became a defect in the composites. When the force exceeded the ultimate bearing strength of the rGO agglomeration, part of the rGO agglomeration fracture. The continuous initiation and expansion of cracks could lead to the ultimate macroscopic destruction of the compacts.

## 3. Experimental Procedure

### 3.1. Preparation of rGO/Fe/Cu Mixture Powders

**Figure 14:**
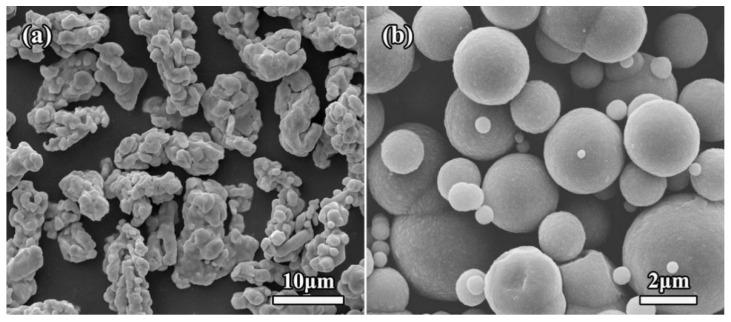
SEM micrographs of the original Cu (**a**) and Fe (**b**) powders.

The raw materials were the dendritic electrolytic copper powders (average size: ~8 μm, >99.9%, Aladdin) and spherical Fe powders (average size: 1~5 μm, >99.9%, Aladdin), as shown in Figure 14. Moreover, graphene oxide (GO) was firstly prepared by a modified Hummers’ method, using the purified natural graphite (99.5%) as the starting materials. Cu and Fe powders were firstly added into the acetone, and then stirred mechanically for 1 h to obtain Cu/Fe mixture powders. The dried Cu/Fe mixture powders were added into this GO aqueous dispersion, and then mechanically stirred to obtain a mixture slurry. After that, the mixed GO/Fe/Cu slurry was freeze-dried to remove the water. Finally, these freeze-dried mixture powders were placed in the hydrazine vapor (90 °C) to reduce GO for the preparation of the rGO/Fe/Cu mixture powders.

### 3.2. Preparation of the Bulk Compacts

The rGO/Fe/Cu mixture powders were primarily cold-compacted in a cylindrical steel die, and then put into the cylindrical graphite dies to sinter under vacuum via a hot-pressed sintering furnace (SXZT-10-10Y). Sintering was carried out at 900 °C for 2 h under an applied load of 35 MPa, with a heating rate of 5 °C/min. In this work, the specimens with nominal composition of Cu–4.0Fe–xrGO (x: 0, 0.1, 0.2 and 0.3 wt.%) were prepared, which were termed as composites 0rGO, 1rGO, 2rGO and 3rGO, respectively. For meaningful comparison, the pure Cu was also prepared using the same processing.

### 3.3. Characterization

X-ray diffraction (XRD, Rigaku D/max-2200, Rigaku corporation, Tokyo, Japan) was performed to determine the phase constitution of the samples using Cu-Kα radiation under vacuum. The rGO was collected by the pickling and centrifugal separation and detected by XRD. Metallographic samples were prepared using conventional grinding and mechanical polishing methods. For observing grain size of Cu matrix, the polished samples were etched by a corrosive liquid (volume ratio, FeCl_3_:HCl:H_2_O = 3:10:100). The morphologies and microstructure of the samples were characterized by optical microscope (OM, Zeiss Axio imager A2m, Carl Zeiss AG, Oberkochen, Germany), transmission electron microscope (TEM, JEM-2100F, JEOL corporation, Tokyo, Japan) and scanning electron microscope (SEM, Hitachi S-3400N, Hitachi limited corporation, Tokyo, Japan; Nova NanoSEM 450, FEI company, Hillsboro, America) equipped with backscattered electron detector (BSE, Oxford corporation, London, England) and energy dispersive X-Ray spectroscopy (EDS, Oxford corporation, London, England). The electric conductivity of all as-sintered compacts was tested using four-point probe (Sigma 2008A, SZJMLY corporation, Shenzhen, China) according to international annealing copper standard (IACS %) [10]. Vickers hardness was evaluated under an applied load of 29.4 N and a load-dwell time of 15 s on the HVS-1000 Vickers sclerometer, and eight indentations were taken for each sample. Compression and tensile tests were carried out on an Instron-8801 universal testing machine under a cross head speed of 1 mm/min at room temperature. The compressive cylinders were 4 mm in diameter and 8 mm in length. The tensile samples were designed to be a gage length of 18 mm and section of 3.5 × 2 mm^2^. The reported results were the average of three measurements. The fracture surface after the tensile tests were observed with SEM.

## 4. Conclusions

Micro/nano hybrid reinforced copper matrix composites with Fe microspheres and rGO nanosheets were fabricated via the vacuum hot-pressing sintering process. For the as-sintered compacts, the micrometer Fe microspheres exhibited the homogeneous dispersion, and the layered G nanosheets exhibited the random distribution in Cu matrix. The rGO nanosheets introduction refined significantly the grains of Cu matrix. The agglomeration tendency of rGO nanosheets increased with the increased content. The hybrid Fe/rGO added composites presented the higher hardness, compressive and tensile σ_0.2_ values compared with pure copper and single Fe-added composite. The as-prepared composites did not rupture under 40% compressive strain and possessed an excellent compressive deformation capability. The higher rGO addition deteriorated UTS and tensile elongation of the composites. The electrical conductivity decreased with the increase of rGO content, but the electrical conductivity still reached more than 33.0% IACS.

## Figures and Tables

**Table 1:** Comparison of electrical conductivity of the as-prepared bulk composites and reported CMCs.

Reinforcements	Matrix	Reinforcements Content (wt.%)	Electrical Conductivity (%IACS)	Preparation Method	Refs.
Fe (0rGO)	Cu	4Fe	36	HPS	This work
Fe + rGO (1rGO)	Cu	4Fe + 0.1rGO	35.3	HPS	This work
Fe + rGO (2rGO)	Cu	4Fe + 0.2rGO	34.2	HPS	This work
Fe + rGO (3rGO)	Cu	4Fe + 0.3rGO	33.8	HPS	This work
Fe	Cu	5Fe	31.0	HPS + HR	[40]
Fe	Cu	10Fe	31.2	Casting	[8]
Fe	Cu	10Fe	25.8	Casting + CR	[8]
Fe	Cu	14Fe	~18	Casting	[9]
Fe	Cu	8.9Fe	33.1	SPS+VHT	[10]
Nb	Cu	13.95Nb	10	MA+UHP	[4]
Nb	Cu	7.15Nb	25	MA+UHP	[5]

HPS: Hot Pressed Sintering, CR: Cold Rolling, SPS + VHT: Spark Plasma Sintering + Vacuum Heat Treatment, MA + UHP: Mechanically Alloying + Uniaxial Hot Pressing.

**Table 2:** Room-temperature tensile properties of the as-prepared bulk compacts and reported CMCs.

Samples	Reinforcements Content	Yield Strength (σ_0.2_, MPa)	Ultimate Tensile Strength (UTS, MPa)	Elongation (*ε*, %)	Preparation Method	Refs.
Pure Cu	--	76.3	207.5	37.1	HPS	This work
Fe/Cu (0rGO)	4Fe (wt.%)	109.2	251.3	25.2	HPS	This work
(Fe+rGO)/Cu (1rGO)	4Fe + 0.1rGO (wt.%)	113.5	210.8	10.2	HPS	This work
(Fe+rGO)/Cu (2rGO)	4Fe + 0.2rGO (wt.%)	134.8	189.8	7.2	HPS	This work
(Fe+rGO)/Cu (3rGO)	4Fe + 0.3rGO (wt.%)	129.4	167.7	3.9	HPS	This work
CF/Cu	0.5CF (wt.%)	55.3	168.7	25.7	HPS	[42]
GNPs/Cu	0.3GNPs (wt.%)	117	172	18	SPS	[32]
GNRs/Cu	0.5GNRs (vol.%)	--	184	65	SPS	[43]

CF: Carbon Fibers, GNPs: Graphene Nanoplates, GNRs: Graphene Nanoribbons; HPS: Hot Pressed Sintering, SPS: Spark Plasma Sintering.

## Data Availability

The date that support the findings of this study are available from the corresponding author upon reasonable request.

## Sample Availability

Samples of the compounds are available from the authors.

## References

[1] Pen H.M., Bai Q.S., Liang Y.C., Chen M.J. (2009). Multiscale simulation of nanometric cutting of single crystal copper—Effect of different cutting speeds. Acta Metall. Sin. Engl. Lett..

[2] Zheng Y.X., Hu P.J., Lv J.F., Xiong H., Lai Z.N. (2021). Separation of arsenic and tin from Cu–As alloy based on phase transformation in a vacuum to form Cu–Fe–S compounds. J. Alloys Compd..

[3] Akbarpour M.R., Mirabad H.M., Alipour S. (2019). Microstructural and mechanical characteristics of hybrid SiC/Cu composites with nano- and micro-sized SiC particles. Ceram. Int..

[4] Botcharova E., Freudenberger J., Schultz L. (2006). Mechanical and electrical properties of mechanically alloyed nanocrystalline Cu–Nb alloys. Acta Mater..

[5] Deng L.P., Han K., Hartwig K.T., Siegrist T.M., Dong L.Y., Sun Z.Y., Yang X.F., Liu Q. (2014). Hardness, electrical resistivity, and modeling of in situ Cu–Nb microcomposites. J. Alloys Compd..

[6] Ohsaki S., Yamazaki K., Hono K. (2003). Alloying of immiscible phases in wire-drawn Cu–Ag filamentary composites. Scripta Mater..

[7] Zhao C.C., Niu R.M., Xin Y., Brown D., McGuire D., Wang E.G., Han K. (2021). Improvement of properties in Cu–Ag composites by doping induced microstructural refinement. Mater. Sci. Eng. A.

[8] Wang M., Jiang Y.B., Li Z., Xiao Z., Gong S., Qiu W.T., Lei Q. (2021). Microstructure evolution and deformation behaviour of Cu-10 wt%Fe alloy during cold rolling. Mater. Sci. Eng. A.

[9] Zou J., Lu D.P., Fu Q.F., Liu K.M., Jiang J. (2019). Microstructure and properties of Cu–Fe deformation processed in-situ composite. Vacuum.

[10] Abbas S.F., Seo S.J., Park K.T., Kim B.S., Kim T.S. (2017). Effect of grain size on the electrical conductivity of copper-iron alloys. J. Alloys Compd..

[11] Yu R.Z., Zhu Z.Y., Li B., Lu Y.Z., Fu B.W., Guan R.G., Lu X. (2021). Performance improvement of laser additive manufactured Cu–Cr alloy via continuous extrusion. J. Alloys Compd..

[12] Wu Z.W., Liu J.J., Chen Y., Meng L. (2009). Microstructure, mechanical properties and electrical conductivity of Cu–12 wt.% Fe microcomposite annealed at different temperatures. J. Alloys Compd..

[13] Song J.S., Hong S.I., Park Y.G. (2005). Deformation processing and strength/conductivity properties of Cu–Fe–Ag microcomposites. J. Alloys Compd..

[14] Liu K.M., Lu D.P., Zhou H.T., Chen Z.B., Atrens A., Lu L. (2013). Influence of a high magnetic fifield on the microstructure and properties of a Cu–Fe–Ag in situ composite. Mater. Sci. Eng. A.

[15] Xie Z.X., Gao H.Y., Wang J., Sun B.D. (2011). Effect of homogenization treatment on microstructure and properties for Cu–Fe–Ag in situ composites. Mater. Sci. Eng. A.

[16] Wu Z.W., Zhang J.D., Chen Y., Meng L. (2009). Effect of rare earth addition on microstructural, mechanical and electrical characteristics of Cu-6%Fe microcomposites. J. Rare Earth..

[17] Song J.S., Hong S.I., Kim H.S. (2001). Heavily drawn Cu–Fe–Ag and Cu–Fe–Cr microcomposites. J. Mater. Process Techig..

[18] Jamwal A., Seth P.P., Kumar D., Agrawal R., Sadasivuni K.K., Gupta P. (2020). Microstructural, tribological and compression behaviour of copper matrix reinforced with Graphite-SiC hybrid composites. Mater. Chem. Phys..

[19] Ramesh C.S., Ahmed R.N., Mujeebu M.A., Abdullah M.Z. (2009). Development and performance analysis of novel cast copper–SiC–Gr hybrid composites. Mater. Des..

[20] Venkatesh R., Vaddi S.R. (2018). Thermal, corrosion and wear analysis of copper based metal matrix composites reinforced with alumina and graphite. Def. Technol..

[21] Liang S.H., Li W.Z., Jiang Y.H., Cao F., Dong G.Z., Xiao P. (2019). Microstructures and properties of hybrid copper matrix composites reinforced by TiB whiskers and TiB_2_ particles. J. Alloys Compd..

[22] Rajkumar K., Aravindan S. (2011). Tribological performance of microwave sintered copper–TiC–graphite hybrid composites. Tribol. Inter..

[23] Rana V., Kumar H., Kumar A. (2021). Fabrication of hybrid metal matrix composites (HMMCs)—A review of comprehensive research studies. Mater. Today Proc..

[24] Guo X.H., Yang Y.B., Song K.X., Li S.L., Jiang F., Wang X. (2021). Arc erosion resistance of hybrid copper matrix composites reinforced with CNTs and micro-TiB_2_ particles. J. Mater. Res. Technol..

[25] Song B., Dong S.J., Coddet P., Zhou G.S., Ouyang S., Liao H.L., Coddet C. (2013). Microstructure and tensile behavior of hybrid nano-micro SiC reinforced iron matrix composites produced by selective laser melting. J. Alloys Compd..

[26] Chen X.R., Fu D.F., Teng J., Zhang H. (2018). Hot deformation behavior and mechanism of hybrid aluminum matrix composites reinforced with micro-SiC and nano-TiB_2_. J. Alloys Compd..

[27] Du X., Skachko I., Barker A., Andrei E.Y. (2008). Approaching ballistic transport in suspended graphene. Nat. Nanotechnol..

[28] McAllister M.J., Li J.L., Adamson D.H., Schniepp H.C., Abdala A.A., Liu J., Herrera-Alonso M., Milius D.L., Car R., Prudhomme R.K. (2007). Single sheet functionalized grapheme by oxidation and thermal expansion of graphite. Chem. Mater..

[29] Lee C., Wei X.D., Kysar J.W., Hone J. (2008). Measurement of the elastic properties and intrinsic strength of monolayer graphene. Science.

[30] Qiao Z.J., Zhou T., Kang J.L., Yu Z.Y., Zhang G.L., Li M., Lu H.M., Li Y., Huang Q., Wang L. (2018). Three-dimensional interpenetrating network graphene/copper composites with simultaneously enhanced strength, ductility and conductivity. Mater. Lett..

[31] Yang Z.Y., Wang L.D., Shi Z.D., Wang M., Cui Y., Wei B., Xu S.C., Zhu Y.P., Fei W.D. (2018). Preparation mechanism of hierarchical layered structure of graphene/copper composite with ultrahigh tensile strength. Carbon.

[32] Shao G.S., Liu P., Zhang K., Li W., Chen X.H., Ma F.C. (2019). Mechanical properties of graphene nanoplates reinforced copper matrix composites prepared by electrostatic self-assembly and spark plasma sintering. Mater. Sci. Eng. A.

[33] Chen F.Y., Ying J.M., Wang Y.F., Du S.Y., Liu Z.P., Huang Q. (2016). Effects of graphene content on the microstructure and properties of copper matrix composites. Carbon.

[34] Liu P.B., Huang Y., Wang L. (2013). A facile synthesis of reduced graphene oxide with Zn powder under acidic condition. Mater. Lett..

[35] Meyer J.C., Geim A.K., Katsnelson M.I., Novoselov K.S., Booth T.J., Roth S. (2007). The structure of suspended graphene sheets. Nature.

[36] Chen L.L., Zhang Z.Y., Huang Y.Y., Cui J.F., Deng Z.X., Zou H.K., Chang K.K. (2019). Thermodynamic description of the Fe–Cu–C system. Calphad.

[37] Zhang J.T., Hao W.X., Lin J.B., Wang Y.H., Chen H.Q. (2020). Effects of carbon element on the formed microstructure in undercooled Cu-Fe-C alloys. J. Alloys Compd..

[38] Wang M., Wang L.D., Sheng J., Yang Z.Y., Shi Z.D., Zhu Y.P., Li J., Fei W.D. (2019). Direct synthesis of high-quality graphene on Cu powders from adsorption of small aromatic hydrocarbons: A route to high strength and electrical conductivity for graphene/Cu composite. J. Alloys Compd..

[39] Belgamwara S.U., Pingaleb A.D., Sharma N.N. (2019). Investigation on electrical properties of Cu matrix composite reinforced by multi-walled carbon nanotubes. Mater. Today Proc..

[40] Wang M., Yang Q.R., Jiang Y.B., Li Z., Xiao Z., Gong S., Wang Y.R., Guo C.L., Wei H.G. (2021). Effects of Fe content on microstructure and properties of Cu−Fe alloy. Trans. Nonferrous Met. Soc. China.

[41] Tan W.Y., Jiang X.S., Shao Z.Y., Sun H.L., Fang Y.J., Shu R. (2022). Fabrication and mechanical properties of nano-carbon reinforced laminated Cu matrix composites. Powder Technol..

[42] Zhang X.J., Yang W.C., Zhang J.Y., Ge X.Y., Liu X.R., Zhan Y.Z. (2019). Multiscale graphene/carbon fiber reinforced copper matrix hybrid composites: Microstructure and properties. Mater. Sci. Eng. A.

[43] Yang M., Weng L., Zhu H., Fan T., Zhang D. (2017). Simultaneously enhancing the strength, ductility and conductivity of copper matrix composites with graphene nanoribbons. Carbon.

[44] Zhang D.D., Zhan Z.J. (2016). Preparation of graphene nanoplatelets-copper composites by a modified semi-powder method and their mechanical properties. J. Alloys Compd..

